# Using on-demand dry ice production as an alternative cryogenic cold chain for bovine artificial insemination outreach in low-resource settings^1^

**DOI:** 10.1093/tas/txaa012

**Published:** 2020-02-03

**Authors:** Mark Kuiper, Maribeth Spencer, Benon M Kanyima, Chin H Ng, Mark Newell, Silver Turyahikayo, Nathaniel Makoni, Damian Madan, Daniel H Lieberman

**Affiliations:** 1 Intellectual Ventures’ Global Good Fund, Bellevue, WA; 2 School of Veterinary and Animal Resources, COVAB, Makerere University, Kampala, Uganda; 3 African Breeders Services Total Cattle Management Limited, Nairobi, Kenya

**Keywords:** artificial insemination, bovine, cryobiology, cryopreservation, dry ice, liquid nitrogen, sperm, semen, storage, theriogenology

## Abstract

Artificial insemination (AI) is widely used in livestock industries to breed for desirable characteristics and increase yields. The standard practice of storing and transporting bovine semen uses liquid nitrogen (LN), a scarce commodity in many regions of the world. This study explored the feasibility of using dry ice, a more readily available alternative. We developed equipment that dispenses dry ice from widely available liquid carbon dioxide (LCO_2_) tanks into an easily transportable device. In vivo fertility results with a dry ice cold chain showed no statistical difference to the conventional LN method. In vitro bovine semen analyses also showed that storage under these conditions minimally affects characteristics associated with fertility. A dry ice cold chain system could leverage the global availability of LCO_2_ to expand the reach of AI and other cold storage applications of biological materials in low-resource settings.

## INTRODUCTION

Over the last few centuries, selective breeding has developed dairy cattle breeds that have optimal productive life and produce large quantities of milk. By contrast, indigenous cows in the developing world survive harsh environments but are far less productive (Kahi and Rewe, 2008; Makoni et al., 2013). Recognizing this discrepancy, many low- and middle-income countries strive to improve beef and milk yields and, therefore, farmers’ incomes and food security by using the genetics of nonendemic breeds. While traditional mating of imported cattle is used to generate both cross and full breeds, artificial insemination (AI) is the prevailing method used to accomplish this goal (van Marle-Köster and Webb, 2014).

Perhaps the greatest breakthrough allowing the expansion of the use of AI came in the 1950s when glycerol was discovered to act as a cryoprotectant for dairy bovine semen (Polge and Rowson, 1952; Bratton et al., 1955). This advance allowed for a greater temporal and, therefore, spatial separation between bovine semen collection and insemination. While other noncryogenic methods exist to store semen, their hold times are too short to be of operational success unless collection and insemination are coordinated.

Initially bovine semen was stored in dry ice or dry ice and ethanol (Foote, 2002; Walters et al., 2009). Subsequent studies showed that liquid nitrogen (LN) provides modestly superior long-term storage (Sherman, 1962; Pickett, 1971; Salisbury and Hart, 1975). For instance, Pickett found that bovine semen stored in LN results in a 2.7% improvement in 60–90-d nonreturn rate (a proxy for conception rate) as compared to dry ice and ethanol (Pickett et al., 1961).

A study that more closely tracked storage duration indicated that dry ice appears equivalent to LN for up to 6 mo, after which bovine semen stored in dry ice loses fertilizing potential (Salisbury and Hart, 1975). These results are likely explained by the hypothesis that, as opposed to dry ice (−78 °C), biological material does not deteriorate in LN (−196 °C) due to the lack of liquid water and insufficient thermal energy to drive chemical reactions (Mazur, 1984).

In developed nations, LN is widely available. Consequently, it is standard practice to store and transport bovine semen in LN or LN vapor. In low-resource settings, however, LN can be unreliable or absent, which can dramatically impede or preclude AI (Makoni et al., 2013; Mwambilwa et al., 2013). Pressurized liquid carbon dioxide (LCO_2_), on the other hand, is often widely distributed in low-resource settings due, primarily, to its use in the carbonated beverage industry (Hayford et al., 2004; Foster, 2008; Linnander et al., 2017). LCO_2_ conversion to dry ice could enable a more widely available and reliable method for preserving and transporting bovine semen.

Here, we describe simple equipment that interfaces with commonly distributed LCO_2_ storage tanks to produce dry ice on demand and a compatible cold storage device capable of storing and transporting frozen bovine semen. We show that the transfer of bovine semen from LN to this dry ice device does not significantly affect sperm viability or acrosome integrity for up to 6 d. Sperm motility does decrease upon initial transfer to dry ice, but no further decline is seen until after dry ice has fully dissipated. Most notably, in a field trial in Uganda, use of the dry ice cold chain showed no significant differences in conception rate when compared to the LN cold chain. Together these results provide foundational justification for a cold chain system that could use dry ice for semen storage and transport when LN is scarce.

## MATERIALS AND METHODS

### Reagents

For in vitro experiments, bovine semen was purchased from Accelerated Genetics (Baraboo, WI) and All West Select Sires, Inc. (Burlington, WA). The purchased bovine semen used in all in vitro experiments were single ejaculates from three Holstein Bulls, diluted in egg yolk-citrate extender and packaged in 0.5-mL French straws. Easy Buffer B and Leja 4-chamber slides were purchased from IMV Technologies (Maple Grove, MN). LCO_2_ was purchased from Airgas (Radnor, PA) and stored in 180-L microbulk tanks from Chart Industries, (Ball Ground, GA). Tanks storage pressure was set to 2,400 kPA. Unless otherwise indicated, all other reagents used in this study were purchased from Sigma (St. Louis, MO).

### Dry Ice Production and Storage Device Prototype

**Figure 1.**
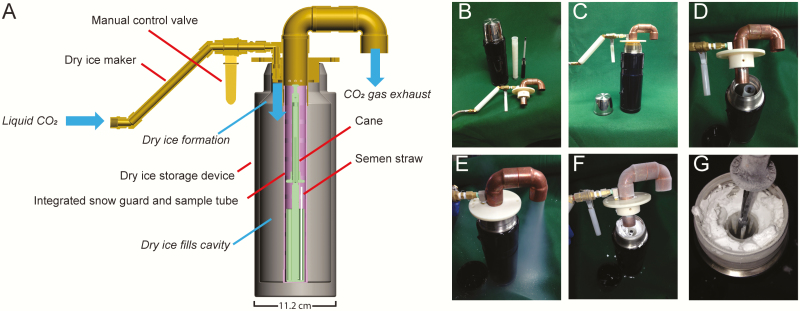
Dry ice cold chain system. (A) The dry ice maker draws liquid CO_2_ from common storage tanks flashing it into dry ice in the vacuum-insulated storage device. Gaseous CO_2_ passes through the integrated snow guard and vents through the exhaust. Straws are kept in a cane within a central tube that doubles as a snow guard. (B) Dry ice vacuum flask is shown with central tube and cane removed and dry-ice-making tool below. (C) Dry-ice-making tool is shown in position to recharge storage device with dry ice. (D–F) The sequence of dry ice refill. (G) Cane raised to the lip of the vacuum flask to remove a straw.

The dry ice storage device consists of a vacuum flask with central tube where bovine semen straws are stored in a custom-designed cane that can be lifted to extract a straw. A King2L vacuum flask (Thermos, Schaumburg, IL) was used as the storage device (Fig. 1). The central tube with 28.6 mm internal diameter was designed to accommodate a single straw-bearing cane and also function as a snow guard (Supplementary Fig. S1). The straw holder (Supplementary Fig. S2) can store up to 21 0.5-mL straws in three partitioned compartments. The device was designed to maintain uniform straw temperatures without the use of an alcohol bath, which was common in previous dry ice storage practices. To extract a straw, the users raised the cane to the lip of the flask (Fig. 1G) and then lowered back down.

We designed a dry ice maker that interfaces with conventional bulk-storage tanks to convert LCO_2_ into dry ice directly (Supplementary Figs S3 and S4) in an off-the-shelf 2-L vacuum-insulated container (Fig. 1D–F). The dry ice maker contains a 1.6-mm-diameter orifice to flash LCO_2_ from a storage tank into dry ice directly inside the 2-L vacuum-insulated storage device. When the manual control valve is opened, dry ice flakes accumulate between the internal wall of the vacuum device and the central tube. Gaseous CO_2_ passes through the central tube pores and vents through the exhaust. The dry ice storage device is then separated from the dry ice maker and covered with a loose-fitting insulated cap.

Where mentioned, a Binder UF V 500 (Binder, Neckarsulm, Germany) freezer set to −80 °C and a Kenmore 253.28042807 (Transform Holdco, Chicago, IL) freezer set to −20 °C were used for in vitro experiments.

### Dry Ice Conversion Efficiency

The percentage of liquid converted to solid was determined by measuring the change in the mass of the liquid in the LCO_2_ microbulk tank using a high-precision platform scale Model PFK988-C600 (Mettler Toledo, Switzerland) and the resulting mass of dry ice produced in the storage device on a desktop scale Model SB8001 (Mettler Toledo, Switzerland). The storage device heat leak was calculated by measuring the mass loss over time using the Model SB8001 scale and multiplying by the carbon dioxide enthalpy of sublimation (571 J/g).

### Temperature Measurements

Three 36 AWG T-type thermocouples (Omega, Stamford, CT) were positioned inside a blank 0.5-mL semen straw (17, 62, and 107 mm from the open end of the straw) filled with epoxy (Loctite Plastic Bonder, Westlake, OH) to secure their position. The instrumented semen straw (Lieberman et al., 2016) was placed next to the bovine semen straws within each device for the duration of the in vitro experiments. Temperatures were logged using a Hobo data logger (Onset, Bourne, MA).

### Sperm Viability and Motility Measurements

**Figure 2.**
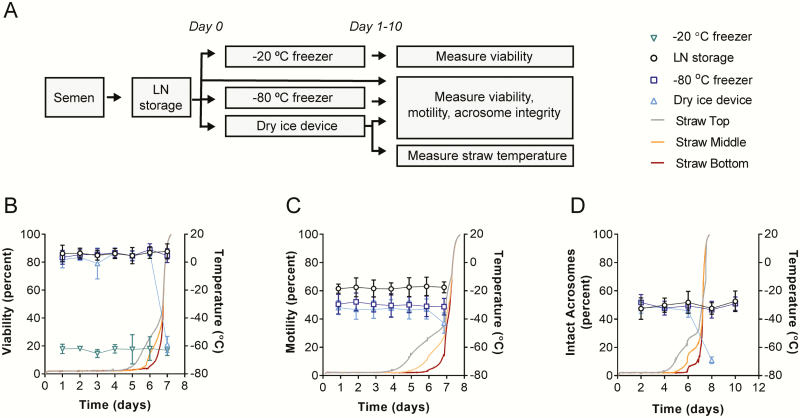
The dry ice cold chain minimally affects bovine semen integrity. (A) Bovine semen straws were transferred from LN to freezers maintained at −80 or −20 °C or the dry ice storage device. At various times, one straw from each of the three bulls was thawed and sperm viability (B) and motility (C) were assessed (*n* = 12: four technical replicates from the same three individual bulls). (D) Acrosome integrity was measured in the semen from two of the bulls (*n* = 4: two technical replicates from each of two bulls). The mean temperatures from the top, middle, and bottom of instrumented bovine semen straws in the cavity of the dry ice storage device are plotted on the right axis (B–D). In all plots, error bars represent SD.

**Figure 3.**
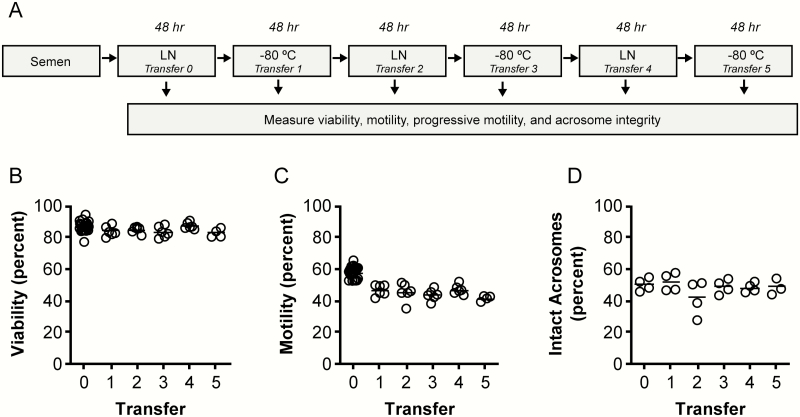
Effects of multiple transfers between LN and −80 °C on bovine semen integrity. (A) Semen straws were transferred between LN and a −80 °C freezer up to five times. Straws were held at each temperature for 48 h. After each transition, straws from each of the three bulls were thawed and sperm viability (B) and motility (C) were measured. Each data point represents the mean of four technical replicates. (D) Acrosome integrity was assessed from two straws at each time point from each of the two bulls.

Bovine semen straws stored in various devices for the times indicated in Figs 2 and 3 were thawed and assessed using the following protocol. All straws were thawed for 1 min in a water bath set at 35 °C. The semen was then transferred to microfuge tubes and incubated in the same bath for an additional 5 min. To assess sperm viability, 20 μL of bovine semen was mixed with 80 μL of 12.5 μg/mL Hoechst 33258 in Easybuffer B. Samples were incubated in the 35 °C bath for 2 min, then 3 μL was loaded into each chamber of the four chamber slides prewarmed to 35 °C. Samples were imaged on a stage warmed to 37 °C in an IVOS II/CASA II computer-assisted sperm analysis instrument (Hamilton Thorne, Beverly, MA) using the default settings on the Animal Motility Viadent package of the Version 1.9 software (1 Hz frame capture speed, 30 frame count, and six fields of view per chamber). For motility assessment, 20 μL of bovine semen was mixed with 80 μL of 25 μg/mL Hoechst 33342 (IDENT) in Easybuffer B. Samples were incubated at 35 °C for 20 min, then 3 μL loaded into each chamber of the four chamber slides prewarmed to 35 °C, and imaged using the default settings on the Animal Motility package of the same instrument (60 Hz frame capture rate, 45 frame count, six fields of view per chamber).

### Acrosome Integrity

For each data point, a minimum of 500 sperm per straw were assessed using previously described methods (Lieberman et al., 2016). Briefly, frozen semen straws were thawed in a water bath set at 35 °C for 30 s. Semen was then transferred to a 1.7-mL microcentrifuge tube and incubated at 35 °C for 1 h. Twenty microliters of semen solution was spread on a microscope slides and air-dried for 15 min. The slides were fixed by immersion in absolute methanol for 15 min. Slides were rinsed in PBS baths twice for 5 min each, transferred to a bath containing 25 μg/mL PNA-FITC (Sigma, St. Louise, MO) for 30 min, and rinsed in three phosphate buffered saline baths for 5 min each. Slides were gently dried using compressed air. One drop of Fluoroshield with DAPI Histology Mounting Medium (Sigma, St. Louise, MO) was then applied to each slide and acrosome integrity was measured using fluorescence microscopy.

### In Vivo Conception Study

Six artificial insemination service providers (AISPs) were enrolled to inseminate cattle in greater Kampala and Mukono districts of Uganda. Dairy cattle were the target animals kept at smallholder farms and managed under best dairy practices, including nutrition, feeding, and maintaining dairy cattle vaccination schedules for East Coast Fever and Foot and Mouth Disease. Farmers provided consent to participate in the study. Cows (*n* = 947) were inseminated between October 2, 2018 and March 1, 2019. This study was approved by the Makerere University School of Veterinary Medicine and Animal Resources Research Ethics Committee—SVAR IACUC/IRB (study SVARREC/07/2018).

Conventional bovine semen was sourced from ABS Global, Inc. (DeForest, WI), frozen using common practices, and shipped to the collection point and subsequently stored in LN or LN vapor. All semen had a sperm cell density of 10 million units per straw and initial motility greater than 55%. Bovine semen was randomly placed within AISPs’ charged LN Dewars or CO_2_ devices.

Each cryogen was alternated every 14 d with half the AISPs using LN and the other LCO_2_ at any given time. LN Dewars were topped up with LN at least once per week and dry ice storage devices were topped up with dry ice at least every third day. Semen inventory was replenished as needed when cryogen was topped up. Semen was maintained within the device for no longer than 14 d. AISPs were trained on the use of the dry ice Dewars handling and insemination practices. Nonreturn rate was determined 21 d (range 18–24 d) following insemination by cow owner or managers assessment. Nonreturn rate, defined as cows not returning to estrus 21 d postinsemination, was evaluated as a binary outcome. A 35-d pregnancy diagnosis follow-up was carried out on a subset of the inseminated cattle that did not return on heat (*n* = 188) by a veterinarian. The pregnancy diagnosis follow-up was randomly assigned to cows that were first sorted by a technician and cryogen. The diagnosis was made using a SonoFarm mini sonogram with a rectal palpation probe (Draminski, Olsztyn, Poland).

AISPs input data associated with insemination and nonreturn status into a custom data collection form generated in Open Data Kit via smartphone and a custom i-cloud data system. Collected data included insemination date and time, client farmer, geographical position system (GPS) location, cow parity or heifer status, body condition score (BCS), cow identification, identification (ID) of the bull served, photograph, and 21-d status. In some cases, not all data fields were recorded.

A body condition score for each cow was determined at each insemination using the BCS Cowdition App (Bayer AG, Leverkusen, Germany). Only cattle with a score between 3.0 and 4.0 were included in the study analysis.

### Statistical Analyses

All data were analyzed in Prism v 7.03 (GraphPad Software Inc., La Jolla, CA) or the R v 3.1.1 statistical environment (R Core Team, 2013). Experiments to evaluate viability, motility, and acrosome integrity of stored sperm in a dry ice device were analyzed using multiple *t-*tests corrected using the Bonferroni–Dunn method to generate multiplicity-adjusted *P* values. The impact of consecutive transfers of bovine semen straws between LN and −80 °C on sperm viability, motility, and acrosome integrity were analyzed using one-way analysis of variance, correcting for multiple comparisons using the Sidak test. A two-sample *t*-test for equality of proportions without continuity correction in the R statistical environment (R Core Team, 2013) was performed on the nonreturn rate and pregnancy diagnosis data sets. Normal-based 95% CIs are used.

## RESULTS

### Performance of Dry Ice Storage Device and Ice Maker Prototypes

Understanding the limitations of unreliable LN networks in low-resource settings, we sought to develop a semen-preserving cold chain system that uses LCO_2_ tanks commonly distributed in low- and middle-income countries. For this study, we developed simple equipment using readily available parts that interfaces with LCO_2_ tanks to produce dry ice on demand and a compatible dry ice storage device capable of holding bovine semen straws for AI outreach (Fig. 1).

To assess the thermal performance of the dry ice storage device, the temperature was measured with instrumented bovine semen straws placed adjacent to bovine semen straws within the cane (Fig. 2B) or attached to the outside of the cane (Fig. 2C and D) within the device cavity. When fully charged with dry ice at approximately 500 kg/m^3^, all portions of the straws held dry ice temperature (~−79 °C) for at least 3.5 d in the laboratory environment (T = 22 °C, range = 19–26 °C). Between 3.5 and 6 d, the bottom of the straws maintained a dry ice temperature, but the middle and tops of the straws warmed (Fig. 2). The dry ice fully dissipated between 6 and 7 d, after which time the cavity of the storage device warmed rapidly to ambient temperature. When charged with 500 kg/m^3^ dry ice, the device averaged a heat leak of 1.6 W in the lab, with extremes of 2.0 and 1.2 W corresponding to full and nearly empty charges, respectively. In the Uganda conception study, the heat leak average was 1.7 W (SD = 0.77 W) translating to 257 g/day (SD = 117 g/day) dry ice sublimation.

The dry ice maker can produce densities of approximately 500 kg/m^3^. If increased hold time is desired, the dry ice density can be increased to 800 kg/m^3^ using a combination of manual compression and refilling with the dry ice maker. The dry ice maker delivered a 30–35% liquid-to-solid conversion efficiency from a 180-L microbulk tank.

### Effects of Storing Bovine Semen in the Dry Ice Device

To assess the effects of storing bovine semen in the dry ice storage device, sperm viability was determined for bovine semen straws transferred from LN to a fully charged dry ice storage device, a −80 °C freezer, or a −20 °C freezer (Fig. 2B). There were no differences in the percentages of viable sperm from straws maintained in LN (−196 °C) or transferred to −80 °C (*P* > 0.99) or the device for up to 6 d (*P* > 0.15). On day 7, the dry ice dissipated in the device, the canister warmed to ambient temperature, and only 21% of the sperm remained viable. Sperm from bovine semen straws transferred from LN to −20 °C significantly lost viability within the first day (*P* < 0.0001) and maintained approximately 20% viability thereafter.

We performed similar experiments to monitor the effects on sperm motility (Fig. 2C). The percentage of motile sperm dropped within the first day after transfer from LN to the −80 °C freezer (−18%, *P* < 0.0004) or the storage device (−22%, *P* < 0.0001). Surprisingly, the percentage of motile sperm stored in the devices was stable thereafter up to 6 d, despite the warming of the top and middle of the straws between 3.5 and 6 d (*P* > 0.073). Motility of sperm stored at −80 °C and in the devices did not differ for 6 d. On day 7, sperm motility decreased concurrent with all regions of the straw warming above −77 °C.

We previously found that sperm acrosome integrity is more sensitive than viability and motility to multiple temperature fluctuations (Lieberman et al., 2016). We sought to determine if acrosome integrity is affected by a single transfer of bovine semen straws from LN to a −80 °C freezer or the dry ice storage device. We measured no differences between the integrity of acrosomes stored in LN versus those from bovine semen straws transferred and held in a −80 °C freezer for up to 10 d (*P* > 0.99) or transferred to and held in the device for up to 6 d (*P* > 0.96). At day 8, bovine semen stored in the device lost acrosome integrity (*P* < 0.0001), coinciding with straws warming to ambient temperature (Fig. 2D).

### Effects of Repeatedly Transferring Bovine Semen Between LN and −80 °C

We next assessed the impacts of multiple shifts between LN and −80 °C. For this experiment, we used an LN Dewar and a −80 °C freezer. Because the thermal performance of the dry ice storage device maintains dry ice temperatures (Fig. 2), a −80 °C freezer served as a suitable proxy. Transferring straws between LN and a −80 °C freezer up to five times did not affect sperm viability (*P* > 0.17; Fig. 3B) or acrosome integrity (*P* > 0.45; Fig. 3D). Sperm motility decreased by approximately 20% upon initial transfer from LN to −80 °C (−19.4%, *P* < 0.001). Subsequent transfers had no further impact on sperm motility (*P* > 0.34; Fig. 3C).

### AI Outcomes

To assess whether the use of the dry ice cold chain for AI outreach affects conception rates, the study carried out an artificial insemination trial in urban and peri-urban Kampala, Mpigi Wakiso, and Mukono districts, Uganda. Over the course of 5 mo, six AISPs performed a total of 948 insemination services, of which 741 collected nonreturn rate data, 392 with LN and 349 with dry ice. The 21-d nonreturn rate for the dry ice cold chain system was 76.5% (95% CI = 71.7%, 80.9%) as compared to 79.1% (95% CI = 74.7%, 83.0%) with the conventional LN cold chain. A two-sample test for equality of proportions without continuity correction indicated that the nonreturn rate difference (2.6%, *P* = 0.45, 95% CI = −3.7%, 8.8%) is not significant.

**Figure 4.**
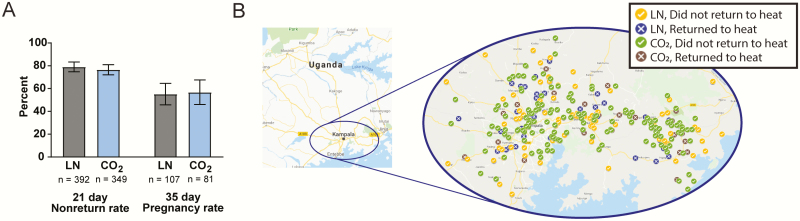
The dry ice cold chain does not affect AI outcomes. (A) AI outcomes for cows inseminated using the conventional LN cold chain and dry ice prototype device (CO_2_) are displayed (error bars represent 95% CI). (B) The geographical locations within Uganda and nonreturn status of cows inseminated with LN and CO_2_ cold chains are shown.

A 35-d pregnancy diagnosis follow-up was carried out using sonography on a subset of randomly selected, inseminated cows that did not return to heat (*n* = 188). Pregnancy was detected in 56.8% (95% CI = 45.3%, 67.8%) and 55.1% (95% CI = 45.2%, 64.8%) of cows inseminated with bovine semen that used the dry ice and LN cold chains, respectively (Fig. 4). A two-sample test for equality of proportions without continuity correction indicated that the pregnancy diagnosis difference (−1.76%, *P* = 0.94, 95% CI = −17.1%, 13.8%) is not significant. We did not observe obvious patterns associated with the outcomes of the different cold chains being affected by insemination location, dam breed, sire breed, or individual inseminators (Fig. 4B; Table 1).

**Table 1.** A breakdown of AI outcomes (21-d nonreturn rate and 35-d pregnancy rate) by type of cold chain, dam breed, sire breed, and artificial insemination service provider

	Dam Breed									
	Friesian		Guernsey		Jersey		Ayrshire		Ankole	Nganda
	Pure	Cross	Pure	Cross	Pure	Cross	Pure	Cross		
Outreach cold chain	NRR (*n*), PR (*n*)	NRR (*n*), PR (*n*)	NRR (*n*), PR (*n*)	NRR (*n*), PR (*n*)	NRR (*n*), PR (*n*)	NRR (*n*), PR (*n*)	NRR (*n*), PR (*n*)	NRR (*n*), PR (*n*)	NRR (*n*), PR (*n*)	NRR (*n*), PR (*n*)
LN	74 (86), 71 (28)	81 (229), 58 (50)	83 (6), 67 (3)	75 (20), 20 (5)	40 (5), 0 (1)	93 (15), 25 (4)	83 (6), 25 (4)	75 (8), 25(4)	100 (2), 0 (1)	73 (15), 0 (2)
CO_2_	75 (75), 52 (23)	81 (171), 56 (32)	91 (11), 50 (4)	67 (24), 17 (6)	50 (10), 50 (4)	56 (9), 67 (3)	67 (3), ND (0)	67 (6), ND (0)	100 (4), 100 (1)	79 (33), 90 (10)
	Sire breed			AISP						
	Ayrshire	Friesian	Jersey	1	2	3	4	5	6	
Outreach cold chain	NRR (*n*), PR (*n*)	NRR (*n*), PR (n)	NRR (*n*), PR (*n*)	NRR (*n*), PR (*n*)	NRR (*n*), PR (*n*)	NRR (*n*), PR (*n*)	NRR (*n*), PR (*n*)	NRR (*n*), PR (*n*)	NRR (*n*), PR (*n*)	
LN	88 (25), 73 (11)	79 (314), 56 (78)	77 (53), 39 (18)	79 (29), 56 (9)	78 (69), 82 (22)	82 (45), 42 (24)	86 (87), 50 (24)	62 (52), 50 (10)	81 (110), 45 (20)	
CO_2_	88 (33), 60 (10)	74 (263), 54 (59)	81 (53), 67 (12)	73 (64), 75 (16)	88 (33), 79 (14)	82 (44), 47 (15)	80 (41), 50 (8)	63 (54), 31 (13)	78 (113), 53 (15)	

Nonreturn rate was defined as cows not returning to estrus 21 d postinsemination, and 35-d pregnancy diagnosis was determined by sonogram with rectal palpation. Pure, pure breed; Cross, cross breed; NRR, percentage of cows not returning to estrus; PR, percentage of pregnant cows; *N*, number of cows considered for analysis; ND, not determined.

## DISCUSSION

LN is the standard medium for storing and transporting bovine semen. However, this cryogenic material is commonly unavailable in times of resource constraints in many countries. In an effort to enable AI in geographies with unreliable LN availability, we conceived of an alternative cold chain that leverages the relatively wide distribution of LCO_2_ due, largely, to the robust infrastructure that supports carbonated beverage manufacture and distribution.

We developed prototypes that interface with commonly used LCO_2_ tanks to dispense dry ice on demand into a transportable storage device. When AISPs in Uganda used this alternative dry ice cold chain, there was no statistical difference in nonreturn or pregnancy rates as compared to the traditional LN cold chain. These results are in accordance with a recent study that reported similar maintenance of fertility when bovine semen was transferred from LN to dry ice (Buranaamnuay et al., 2016) and with earlier studies that compared LN to dry ice with an alcohol bath (Sherman, 1962; Foote, 2002; Walters et al., 2009). This is the first study, to the authors’ knowledge, to test and measure nonreturn and pregnancy rates of a dry ice storage system without an alcohol bath.

The flask used in the current study has a 6-d hold time, which may be insufficient for some AISPs. Significant opportunity exists to design dry ice equipment capable of storing more products, products with different dimensions, and improving hold times. For instance, a two- to three-factor improvement on hold time is possible by increasing the dry ice density to commercial grade (~1,500 kg/m^3^) or improving the thermal performance of the storage device.

Observations of in vitro characteristics of bovine semen quality are consistent with the dry ice cold chain maintaining bovine semen fertility. In this study, we observed that when bovine semen is stored within the storage device, sperm viability and acrosome integrity are maintained for 6 d, which is consistent with previous work (Pickett et al., 1961; Salisbury and Hart, 1975) that demonstrated viability for up to 6 mo on dry ice. We also found that sperm motility decreases by approximately 20% upon initial transfer to dry ice and is then sustained until dry ice dissipates and the temperature of the storage device increases dramatically. A limitation of this analysis is that we measured gross motility and not more nuanced kinematic motility measurements, which can be more predictive of fertility (Rodriguez-Martinez, 2003). Nevertheless, we did measure nonreturn and pregnancy rates as more direct indicators to support the findings of this study.

At first glance, the gross motility decreases do not appear to be consistent with nonreturn results. That is, if motility decreases by 20%, should nonreturn and pregnancy rates also decrease? The observation that conception was unaffected suggests that the transfer of bovine semen from LN to dry ice temperatures primarily affects the motility of a subpopulation of sperm that does not contribute to fertility.

Experiments where we repeatedly transferred bovine semen between −196 and −80 °C reinforce this inference. If cryogenic damage indiscriminately affects sperm upon transitioning between −196 and −80 °C, then a stepwise decrease in motility would be expected to accompany repeated transfers. We measured motility decreases only after the initial transfer to −80 °C. The observation that motility was consistent thereafter suggests that susceptible and more robust sperm populations exist within the same bovine semen straw.

While repeatedly transferring bovine semen from LN to dry ice temperatures did not deleteriously affect in vitro characteristics of bovine seminal biology, these measurements are imprecise predictors of fertility (Rodriguez-Martinez, 2003, 2006; Oliveira et al., 2013). As such, we hesitate to recommend this practice in the absence of in vivo experiments that would more accurately quantify the risks. However, this aspect would benefit from an additional study as it may occur in practice.

Data in this study provides foundational support for the transfer of bovine semen from LN to a dry ice cold chain for limited durations. If LN is available, then bovine semen freezing, storage, and transport should continue to be performed using conventional protocols. If LN is unavailable, our data show that bovine semen can be transferred to a dry ice cold chain for short duration storage and/or transportation with minimal, if any, effects on fertility.

Based on these findings, we expect that this alternative cold chain could enable greater geographical outreach of AI. The system also has the potential to support the delivery of other biological materials that are transported in LN in low-resource settings, such as East Coast Fever stabilate (Perry, 2016). Similarly, the ability to make dry ice on demand from commonly distributed LCO_2_ tanks could enable the collection, storage, and transportation of various labile biological materials from remote settings to more centralized testing facilities. Additional studies are needed to evaluate how to operationalize this system and evaluate the economics of this approach.

## SUPPLEMENTARY DATA

Supplementary data are available at Translational Animal Science online.

S1 Fig. Schematic of the central tube with list of materials.

S2 Fig. Schematic of the straw holder with list of materials.

S3 Fig. Schematic of the dry ice producing adaptor with list of materials.

S4 Fig. Schematic of the top cover for the dry ice producing adaptor.

txaa012_suppl_Supplementary_Figure_S1

txaa012_suppl_Supplementary_Figure_S2

txaa012_suppl_Supplementary_Figure_S3

txaa012_suppl_Supplementary_Figure_S4

## References

[CIT0001] Bratton R. W., Foote R. H., and Cruthers J. C.. 1955 Preliminary fertility results with frozen bovine spermatozoa. J. Dairy Sci. 38:40–46.

[CIT0002] Buranaamnuay K., Seesuwan K., and Saikhun K.. 2016 Preliminary study on effects of bovine frozen semen storage using a liquid nitrogen-independent method on the quality of post-thaw spermatozoa. Anim. Reprod. Sci. 172:32–38. 27421230 10.1016/j.anireprosci.2016.06.011

[CIT0003] Foote R. H 2002 The history of artificial insemination: selected notes and notables. J. Anim. Sci. 80:1–10.

[CIT0004] Foster R. J 2008 Coca-globalization: following soft drinks from New York to New Guinea. New York (NY): Palgrave Macmillan.

[CIT0005] Hayford K., Privor-Dumm L., and Levine O.. 2004 Improving access to essential medicines through public-private partnerships. Baltimore (MD): International Vaccine Access Center.

[CIT0006] Kahi A. K., and Rewe T. O.. 2008 Biotechnology in livestock production: overview of possibilities for Africa African. J. Biotechnol. 7:4984–4991.

[CIT0007] Lieberman D., McClure E., Harston S., and Madan D.. 2016 Maintaining semen quality by improving cold chain equipment used in cattle artificial insemination. Sci. Rep. 6:28108. 27313137 10.1038/srep28108 PMC4911568

[CIT0008] Linnander E., Yuan C. T., Ahmed S., Cherlin E., Talbert-Slagle K., and Curry L. A.. 2017 Process evaluation of knowledge transfer across industries: leveraging Coca-Cola’s supply chain expertise for medicine availability in Tanzania. PLoS One 12:1–16. 10.1371/journal.pone.0186832 PMC5679563 29121051

[CIT0009] Makoni N., Mwai R., Redda T., van der Zijpp A., and van der Lee J.. 2013 White gold: opportunities for dairy sector development collaboration in East Africa. Centre for Development Innovation, Wageningen, The Netherlands p. 203.

[CIT0010] Mazur P 1984 Freezing of living cells: mechanisms and implications. Am. J. Physiol. 247:C125–C142. 6383068 10.1152/ajpcell.1984.247.3.C125

[CIT0011] Mwambilwa K., Yambayamba K. E., and Simbaya J.. 2013 Evaluation of the reproductive performance and effectiveness of artificial insemination on smallholder dairy farms in Zambia location of study. J. Agric. Sci. 3:391–400.

[CIT0012] Oliveira L. Z., Monteiro F. M., Carla E., and Celeghini C.. 2013 The importance of semen quality in AI programs and advances in laboratory analyses for semen characteristics assessment. In: Lemma A., editor. Success in artificial insemination—quality of semen and diagnostics employed. London (UK): InTech p. 1–16.

[CIT0013] Perry B. D 2016 The control of East Coast fever of cattle by live parasite vaccination: a science-to-impact narrative. One Health. 2:103–114. 28616483 10.1016/j.onehlt.2016.07.002 PMC5441314

[CIT0014] Pickett B. W 1971 Factors affecting the utilization of frozen bovine semen for maximum reproductive efficiency. AI Digest. 19:8.

[CIT0015] Pickett B. W., Martig R. C., and Cowan W. A.. 1961 Preservation of bovine spermatozoa at −79 and −196° C. J. Dairy Sci. 44:2089–2096.

[CIT0016] Polge C., and Rowson L. E. A.. 1952 Fertilizing capacity of bull spermatazoa after freezing at -79 C. Nature 169:626–627. 10.1038/169626b0 14929257

[CIT0017] R Core Team 2013 R: a language and environment for statistical computing. R Foundation for Statistical Computing, Vienna, Austria.

[CIT0018] Rodriguez-Martinez H 2003 Laboratory semen assessment and prediction of fertility: still utopia ? Reprod. Domest. Anim. 318:312–318. 10.1046/j.1439-0531.2003.00436.x 12887570

[CIT0019] Rodrıguez-Martinez H 2006 Can we increase the estimative value of semen assessment. Reprod. Domest. Anim. 41:2–10. 16984464 10.1111/j.1439-0531.2006.00764.x

[CIT0020] Salisbury G. W., and Hart R. G.. 1975 Functional integrity of spermatozoa after storage. BioScience 25:159–165. 1140493

[CIT0021] Sherman J. K 1962 Preservation of bull and human spermatazoa by freezing in liquid nitrogen vapour. Nature 194:1291–1292.

[CIT0022] van Marle-Köster E., and Webb E. C.. 2014 A perspective on the impact of reproductive technologies on food production in Africa. In: Lamb G. and DiLorenzo N., editors. Current and future reproductive technologies and world food production. Advances in Experimental Medicine and Biology. vol. 752 New York (NY): Springer; p. 199–211. 10.1007/978-1-4614-8887-3_10 24170361

[CIT0023] Walters E. M., Benson J. D., Woods E. J., and Critser J. K.. 2009 The history of sperm cryopreservation. In: Pacey A. A. and Tomlinson M. J., editors. Sperm banking: theory and practice. Cambridge University Press, Cambridge, UK p. 2–10.

