# Supplementary figures and images for: Using on-demand dry ice production as an alternative cryogenic cold chain for bovine artificial insemination outreach in low-resource settings
Source: Transl Anim Sci. 2020 Feb 3;4(2):1196–205. doi: 10.1093/tas/txaa012 (PMC7201085; doi:10.1093/tas/txaa012)

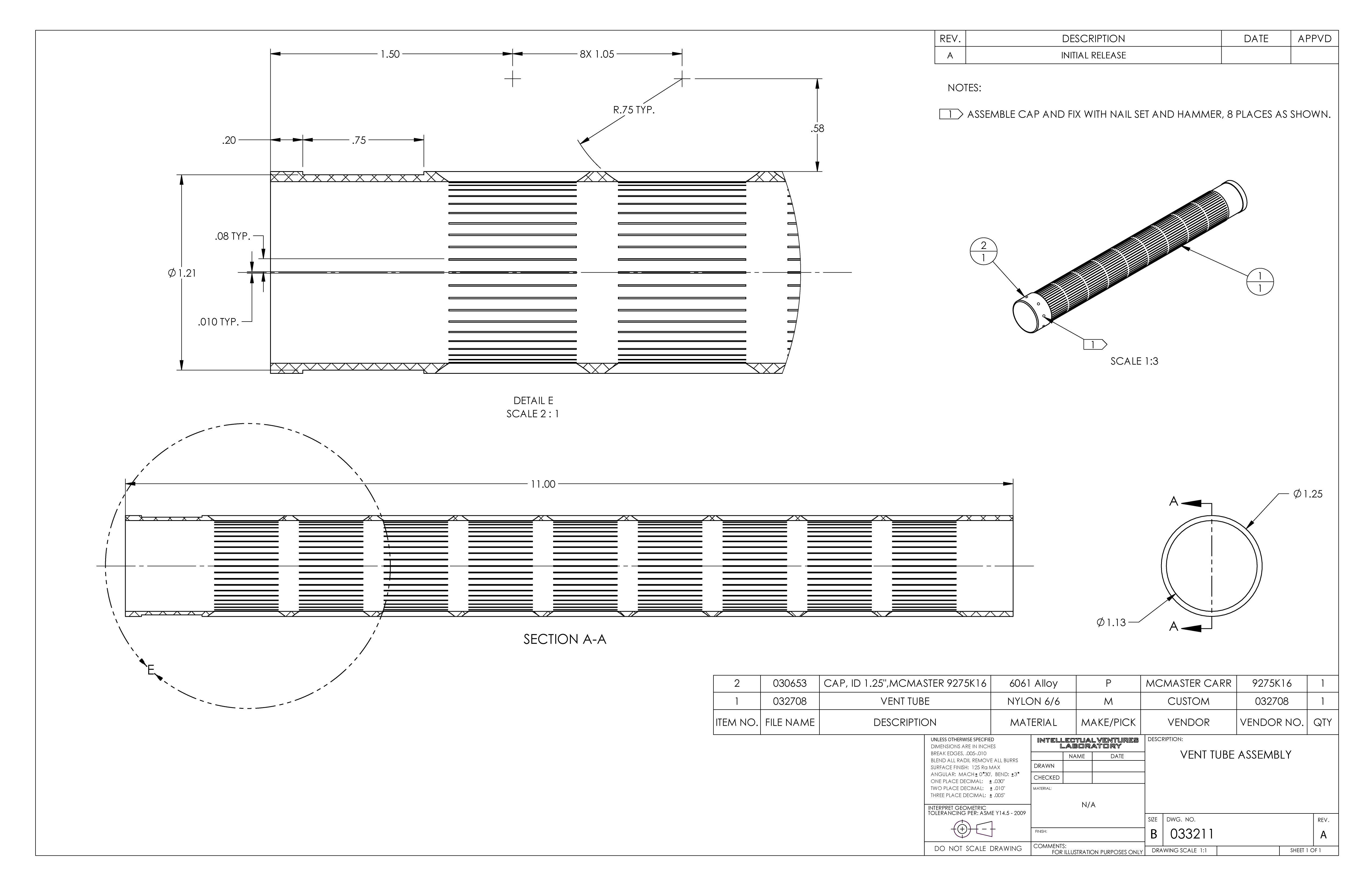

Supplement: txaa012_suppl_Supplementary_Figure_S1 [file txaa012_suppl_supplementary_figure_s1.png]

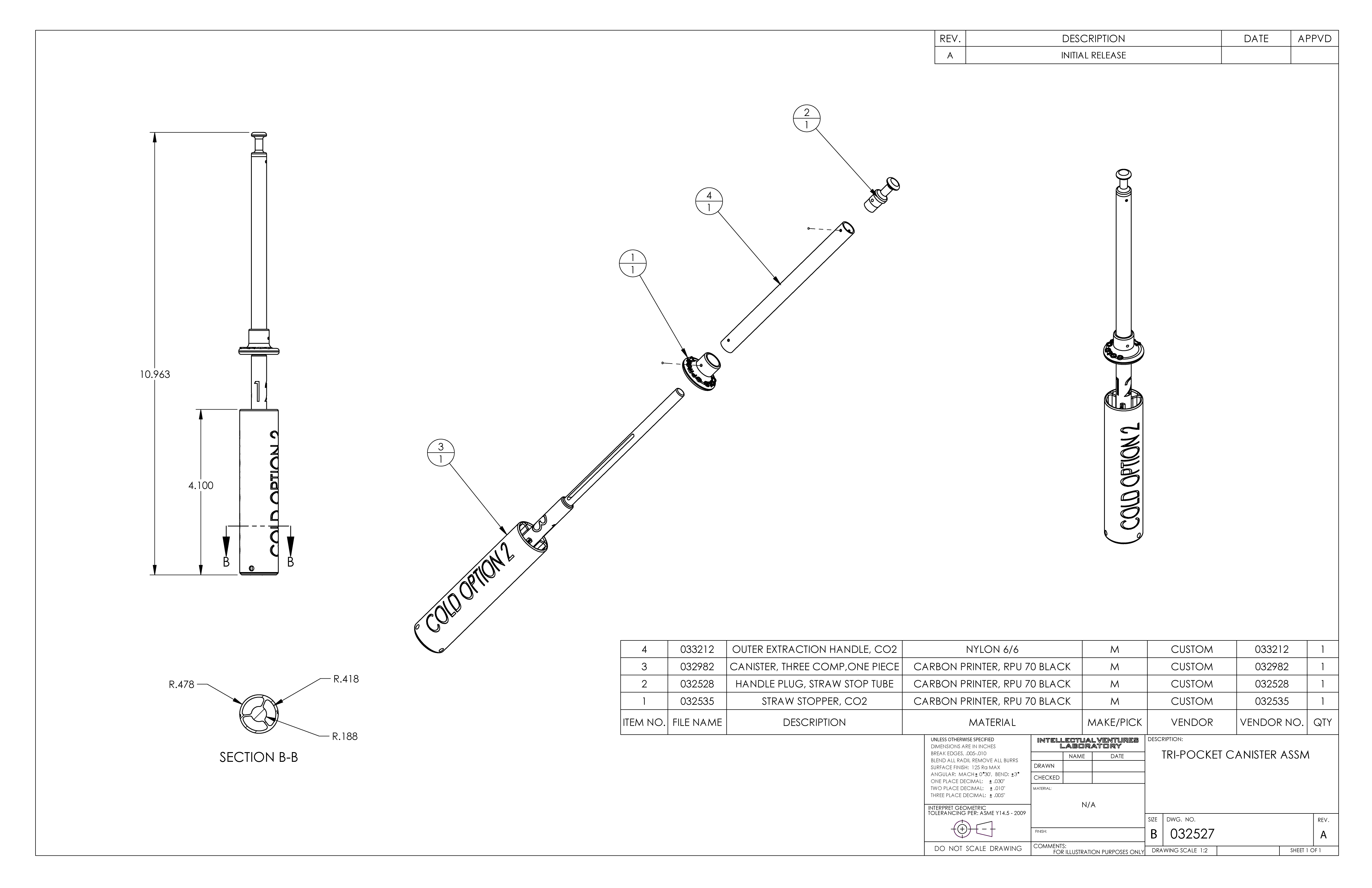

Supplement: txaa012_suppl_Supplementary_Figure_S2 [file txaa012_suppl_supplementary_figure_s2.png]

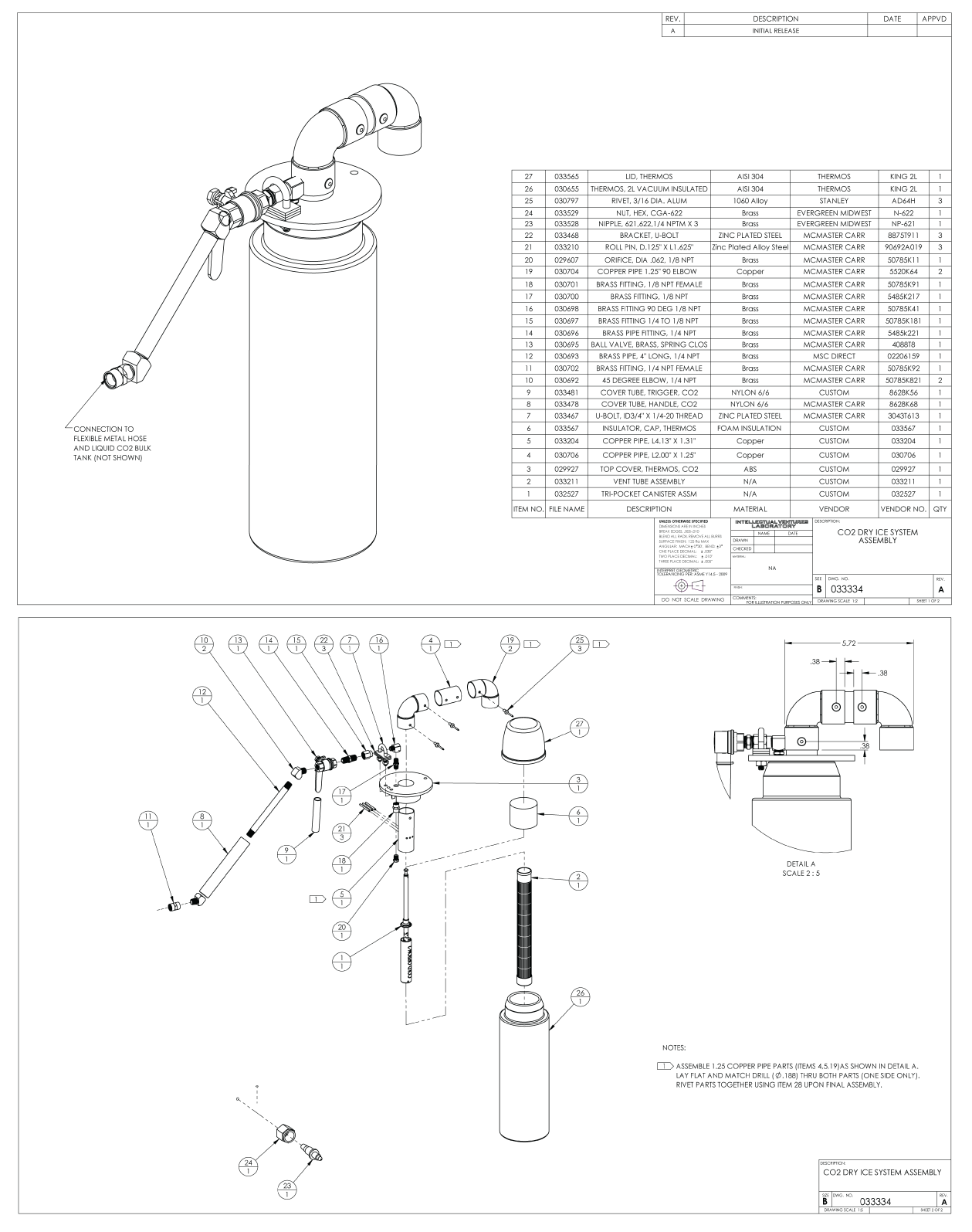

Supplement: txaa012_suppl_Supplementary_Figure_S3 [file txaa012_suppl_supplementary_figure_s3.png]
